# Bilateral spontaneous quadriceps tendon rupture: a case report and literature review

**DOI:** 10.1051/sicotj/2023031

**Published:** 2023-11-03

**Authors:** Mohammad Alkhatatba, Yazan Anaqreh, Suhaib Bani Essa, Ala’a Alma’aiteh, Hamzeh Ziad Audat, Naser Obeidat, Marwan Ahmed

**Affiliations:** 1 Assistant Professor Division of Orthopedics, Department of Special Surgery, Faculty of Medicine, Jordan University of Science and Technology Irbid 22110 Jordan; 2 PGY-4 orthopedic resident Division of Orthopedics, Department of Special Surgery, Faculty of Medicine, Jordan University of Science and Technology Irbid 22110 Jordan; 3 Department of Pediatric and Neonatology, Faculty of Medicine, Hashemite University Zarqa Jordan; 4 Medical Student, Faculty of Medicine, Jordan University of Science and Technology Irbid 22110 Jordan; 5 Assistant Professor of Radiology, Department of Diagnostic Radiology and Nuclear Medicine, Faculty of Medicine, Jordan University of Science and Technology Irbid 22110 Jordan; 6 PGY-5 orthopedic resident Division of Orthopedics, Department of Special Surgery, Faculty of Medicine, Jordan University of Science and Technology Irbid 22110 Jordan

**Keywords:** Bilateral, Quadriceps tendon rupture, Spontaneous, Diagnosis, Surgical repair, Rehabilitation

## Abstract

Bilateral spontaneous quadriceps tendon rupture is a rare condition characterized by the simultaneous tear of the fibrous tissue connecting the quadriceps muscle to the patella bone. Prompt diagnosis is crucial for appropriate treatment and optimal outcomes. We present a case of a 70-year-old male with bilateral knee pain and an inability to walk, resulting from a trivial fall. Despite initial misdiagnosis, a thorough evaluation, including physical examination and imaging, revealed bilateral quadriceps tendon rupture. Surgical repair was performed, followed by a comprehensive rehabilitation program. At the four-month follow-up, the patient showed significant improvement in pain and function. This article provides a comprehensive review of the existing literature on bilateral quadriceps tendon rupture, emphasizing the challenges in the diagnosis and management of this rare condition. Early diagnosis, prompt surgical intervention, and a tailored rehabilitation program are crucial for successful outcomes.

## Introduction

Bilateral spontaneous quadriceps tendon rupture is a rare but physically exhausting condition that can cause difficulties in movement and stability. This condition occurs when the fibrous tissue connecting the quadriceps muscle to the patella bone tears simultaneously. The tensile strength of the quadriceps tendon is related to its thickness, making it unlikely to be injured following minor trauma without preexisting degeneration. The diagnosis of bilateral simultaneous rupture of the quadriceps tendon can be challenging due to its rarity, and a timely diagnosis is essential for appropriate treatment and optimal clinical outcomes. In this article, we will be presenting a case managed at our institution, as well as a comprehensive review of the existing literature on the topic.

## Case report

**Figure 1:**
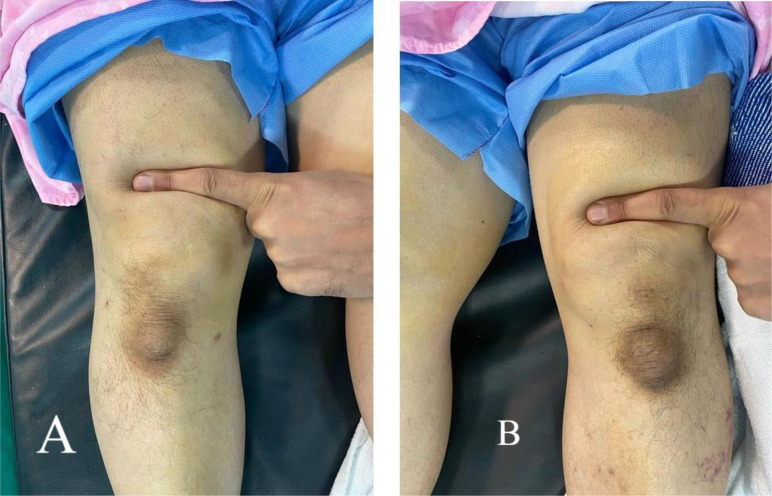
Preoperative examination showing suprapatellar gap. (A) Right knee, (B) left knee.

A 70-year-old male patient presented to our clinic with bilateral knee pain and an inability to walk. The patient reported that his chief complaint had surfaced three weeks before the clinic appointment, after a seemingly trivial fall from a standing height. During the fall, the patient landed on his back with his knee flexed, rendering him unable to stand. At the time of injury, the patient promptly sought medical attention at a primary care facility, where he received analgesics and was referred to our orthopedic clinic as a case of knee osteoarthritis. The patient’s medical history was unremarkable, with no previous history of medication intake or prodromal knee pain before the injury. Despite his advanced age, the patient was a physically active individual who routinely engaged in physical exercise. Upon conducting a clinical examination, we identified a palpable gap in the suprapatellar region and bilateral loss of knee extension, as depicted in Figure 1.

**Figure 2:**
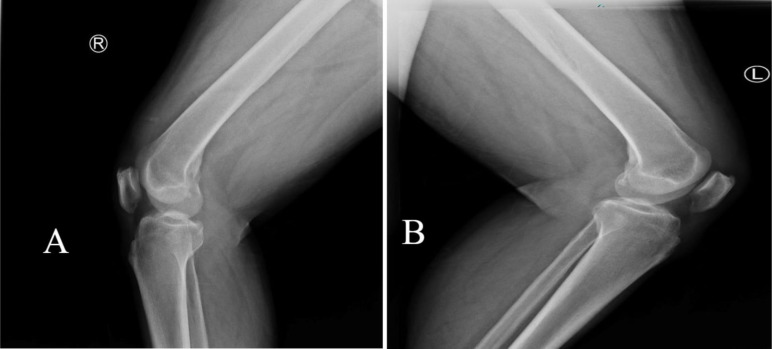
Pre-operative X-ray. (A) Right lateral view and (B) left lateral view.

**Figure 3:**
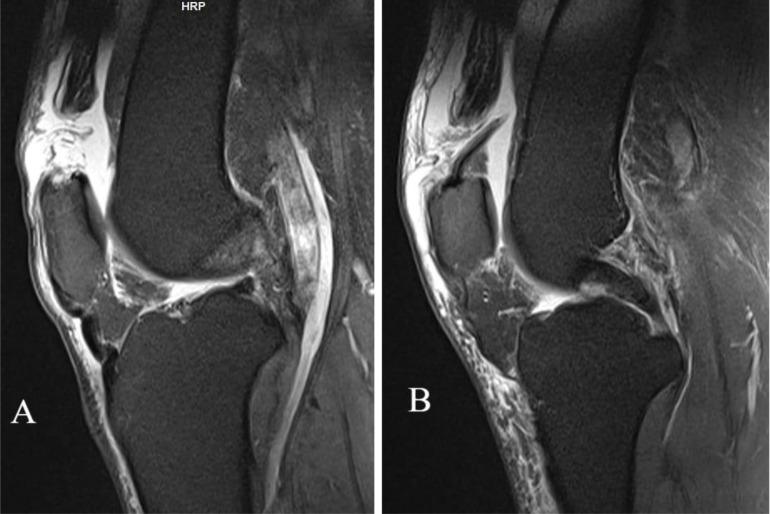
MRI knee showing bilateral rupture of the quadriceps tendon, as there is a noticeable increase in signal intensity on both sides along with loss of fiber continuation. (A) Right knee MRI; (B) left knee MRI.

An X-ray of the patient’s knee was obtained, which shows bilateral patella infera, as depicted in Figure 2. However, a magnetic resonance imaging (MRI) study revealed bilateral quadriceps tendon rupture, as shown in Figure 3.

The results of the laboratory analysis indicated that the individual’s kidney profile, lipid profile, Hemoglobin A1c(6.1), serum uric acid, parathyroid hormone, vitamin D, serum calcium, and serum phosphorus were found to be within the normal range. Additionally, the rheumatology workup did not show any abnormalities. The individual’s body mass index (BMI) was recorded as 32, which falls under the category of obesity class 1 as per the standard BMI classification.

Dual Energy X-ray Absorptiometry (DEXA) scan for osteoporosis was normal with a total *Z*-score at the lumbar area 1.6.

**Figure 4:**
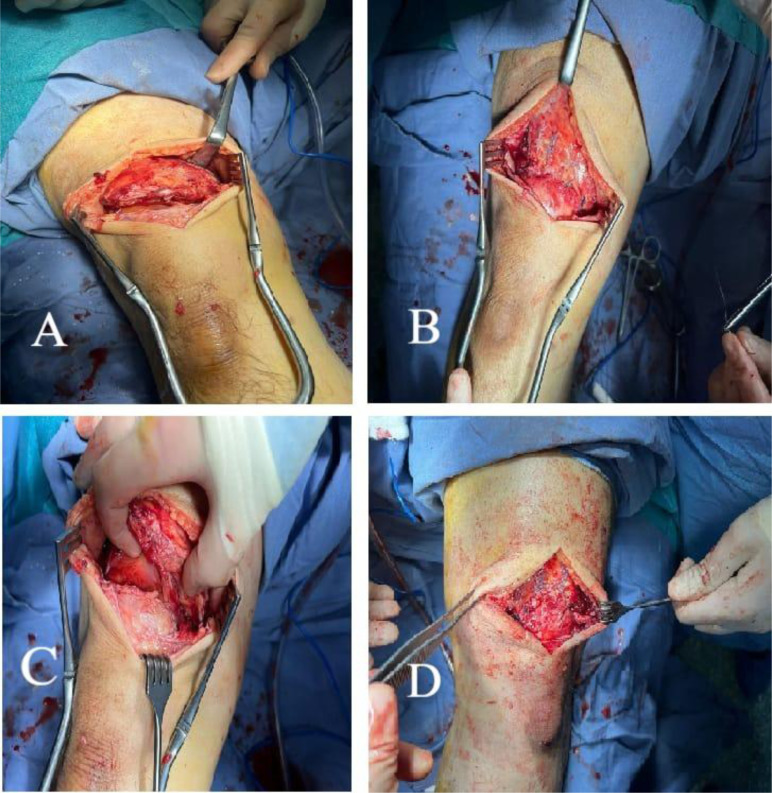
Intraoperative imaging showing complete tear of degenerative right quadriceps tendon edges (A), an approximation of the tendon to the patella following repair using suture anchor and Krackow suture technique (B), near complete tear of the left quadriceps tendon (B), and repair of the quadriceps tendon using anchor sutures (D). Following the surgery, the patient was put on a comprehensive rehabilitation program, which included immobilization in a hinge knee brace locked in extension for 1 month, along with non-weight-bearing exercises. The patient’s rehabilitation program progressed to involve a gradual increase in passive flexion for the second month until he reached 90° of flexion.

Following a thorough evaluation of the patient’s condition, a bilateral primary tendon repair procedure was carried out. This involved the careful debridement of the distal stump of the quadriceps tendon, which exhibited signs of fragility and degeneration, and measured approximately 1 cm in length from the superior pole of the patella. Subsequently, the proximal stump of the tendon was reattached to the superior pole of the patella using two anchor sutures, each 4.75 mm the size, as depicted in the accompanying Figure 4.

Following the surgery, the patient was put on a comprehensive rehabilitation program, which included immobilization in a hinge knee brace locked in extension for 1 month, along with non-weight-bearing exercises. The patient’s rehabilitation program progressed to involve a gradual increase in passive flexion for the second month until he reached 90° of flexion.

**Figure 5:**
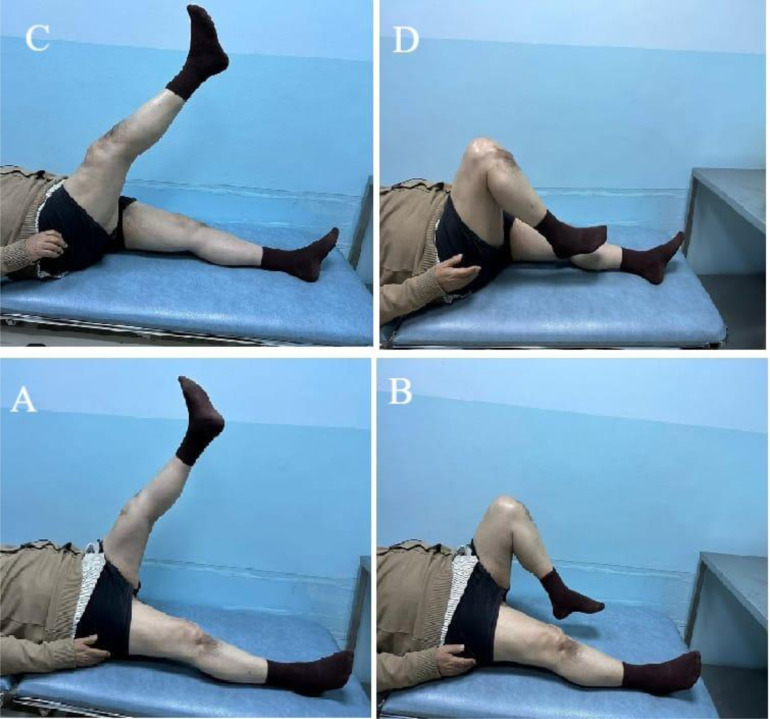
Knee range of motion at 4 month follow-up. (A) Left knee extension, (B) flexion, (C) right knee extension, (D) flexion.

In the third month, he began a gradual increase in active range of motion exercises. In the final month leading up to the final follow-up, he commenced quadriceps muscle strengthening exercises. At the four-month follow-up, the patient reported significant improvement in knee pain and function. He was able to stand and walk and return to his normal daily activities, albeit with minor limitations in certain activities requiring more knee strength and stability (Figure 5).

## Discussion

Bilateral spontaneous quadriceps tendon (QT) rupture is a rare phenomenon, as indicated by a UK study reporting an incidence rate of 1.37 per 100,000 individuals annually [1]. The study revealed a mean age of 50.5 years for men and 51.7 years for women, with a higher occurrence noted among males [2]. Another investigation highlighted that atraumatic simultaneous bilateral quadriceps tendon rupture accounts for 30%–35% of all cases, underscoring the uncommon nature of this condition [3].

It is a physically exhausting condition that occurs when the fibrous tissue connecting the quadriceps muscle to the patella bone tears simultaneously. The quadriceps tendon is responsible for transferring the force generated by the quadriceps muscle to the knee joint, which enables movement and provides stability.

The tensile strength of the quadriceps tendon is related to its thickness with a study showing that a quadriceps tendon with a cross-sectional area of 65 mm^2^ has a tensile stress value of 37 N/mm^2^ [4]. With such an exceedingly powerful structure, it’s unlikely to be injured following a minor trauma without preexisting degeneration. This has been shown by animal studies, proposing that about 50%–75% of the fibers of the quadriceps need to be damaged before a complete rupture under a physiological load occurs [5].

Recent academic inquiries have shed light on the intricate vascularization patterns of the quadriceps tendon, positing the involvement of vascular imbalances in the genesis of ruptures. The quadriceps tendon typically receives vascular supply from three distinct systems: the medial arcade, nourishing its medial edge; the lateral arcade, supplying its lateral border; and the peripatellar vascular ring, an elaborate vascular network enriching the tendon’s distal 1 cm. The tendon was further categorized into three zones, Zone 1 is located between 0 and 1 cm from the superior pole of the patella, Zone 2 lies between 1 and 2 cm from the superior pole of the patella, and Zone 3 is located >2 cm from the superior pole of the patella. Zone 2 was found to be less vascular than Zones 1 and 3. This disparity can be attributed to the peripatellar ring supplying Zone 1 and the medial and lateral arcades, with a plentitude of vessels at the musculotendinous junction, catering to Zone 3 [6, 7]. Additionally, observations on the sagittal plane disclosed varied vascular densities, with the superficial plane of the rectus femoris displaying higher vascularity than the middle unit (the convergence of tendinous portions of the vastus medialis and vastus lateralis) and the deepest plane (vastus intermedius tendon) [8]. Hence, these observations led to the hypothesis that compressive forces exerted by the femoral condyles on the articular side of the tendon could cause reduced vascularity in its deepest segment, thereby increasing the predisposition to tendon ruptures [9] which was shown to be the most common site of rupture in several studies [10].

Many conditions have been implicated as predisposing factors for tendon rupture, altering the tendon ultrastructure or impacting its vascularity [11]. These conditions encompass a range of medical issues such as chronic renal failure, hyperparathyroidism [12], diabetes, systemic lupus erythematosus [13], and the chronic use of specific medications including steroids, quinolones, statins, anabolic steroids, and intranasal steroids [14, 15]. It is of paramount importance to remain vigilant for these established causes, especially in patients younger than 50 years of age. Research indicates that younger patients experiencing bilateral quadriceps tendon rupture are statistically more likely to have an underlying medical pathology than their older counterparts [3].

However, it is noteworthy that cases of quadriceps tendon rupture occurring without any discernible risk factors are exceptionally rare. To highlight this rarity we have curated a detailed table summarizing cases reported in the literature where quadriceps tendon rupture occurred in the absence of any identifiable risk factors (Table 1).

**Table 1:** Summary for cases reported in the literature where bilateral quadriceps tendon rupture occurred in absence of any identifiable risk factors.

Authors	Age/sex	Mechanism of injury	Risk factor	Initial diagnosis	Diagnosis delay	Surgical technique	Outcome
Wetzler and Merkow 1950 [41]	46/Male	Spontaneous	None	Neurologic condition	42 days	Transosseous Fixation	Full recovery
Kleintz et al. [42]	59/Male	Spontaneous	None	Ligamentous injury	14 days	Primary repair	Full recovery
Sagiv et al. [43]	78/Male	Spontaneous	None	Neurologic condition	3 days	Nonabsorbable sutures to the patella using drill holes in its upper pole	Full recovery
Calvo [14]	39/Male	Spontaneous	None	No initial diagnosis	21 days	Left tendon was repaired with wire sutures passed through drill holes in the patella. End-to-end repair was carried out in the right tendon.	Full recovery
Onuoha et al. [44]	60/Male	Spontaneous	None	Diagnosed at first presentation	None	Primary repair	Full recovery

In light of the rarity of these injuries, a timely diagnosis can be challenging for the treating physician with misdiagnosis at initial presentation reported to occur in 67% of patients [16]. This can be attributed to obesity which can be a significant factor in the delayed diagnosis of bilateral simultaneous rupture of the quadriceps tendon, as it enlarges the soft tissue envelope and can obscure the suprapatellar gap [17]. In addition, misdiagnoses can occur in the elderly population and contributing factors include medical conditions such as strokes, occult fractures, rheumatoid arthritis, bilateral effusion, and others, which may lead to difficulty in leg movement and, consequently, hinder the ability to perform an appropriate extensor mechanism examination [18].

The inability to detect the injury at the time of initial presentation can lead to a delay in providing appropriate treatment, which in turn can result in suboptimal clinical outcomes [19]. Thus, it is imperative for the attending physician to conduct a comprehensive examination, including a detailed medical history and physical examination, and to supplement it with relevant imaging, if necessary. The history should include a detailed inquiry into the mechanisms of the injury, any pre-existing medical conditions, and the use of steroids, as these can provide key diagnostic insights. In the physical examination, the presence of knee pain, effusion, and palpable suprapatellar gap, which can be further emphasized with active quadriceps muscle contraction as hemarthrosis can obscure the gap [20], along with extensor mechanism insufficiency are critical diagnostic clues [21, 22]. However, in some cases, the presence of intact medial and lateral retinacula results in a misleading diagnosis of an intact extensor mechanism [23]. Nevertheless, all of these factors remain valuable diagnostic indicators.

Imaging can be employed to validate a diagnosis, and in case of doubt, plain films, ultrasound (US), and MRI are effective and viable options. Plain films are easily accessible in emergency settings and are typically obtained as a standard diagnostic measure to eliminate potential differential diagnoses, such as fractures, especially patellar fractures. Careful observation of subtle findings on the plain films, such as soft tissue defects, and avulsed bone fragments at the distal end of the quadriceps muscle, patella baja, knee effusion, and the presence of patellar spurs which was reported as a risk factor [21] can facilitate the accurate diagnosis of quadriceps tendon rupture [24–26]. Ultrasonography, despite being more cost-effective and accessible than MRI, is susceptible to operator dependence and the risk of missed diagnoses if performed by an inexperienced sonographer [27]. MRI is the preferred diagnostic modality for quadriceps tendon rupture, as it boasts 100% sensitivity and specificity, with a positive predictive value of 100 compared to ultrasound [16]. However, MRI’s higher cost and limited accessibility in emergency settings pose significant limitations.

The management strategy primarily involves reattaching the ruptured tendons to the patella. Various surgical techniques have been delineated for primary repair: direct end-to-end suture repair [28] is recommended for midsubstance tears, whereas for ruptures occurring near or at the osseotendinous junction, options include patellar drill holes or anchor sutures [5]. The latter method offers the advantages of smaller incisions and reduced operating time in comparison to patellar drill holes.

Despite biomechanical and clinical studies indicating no significant disparities between these techniques, it is essential to highlight the scarcity of substantial data from extensive series or comparative studies in the existing literature. This scarcity hampers the ability to conclusively affirm these findings [29].

Various other techniques have been described, particularly for chronic ruptures with tendon retraction and a considerable gap between the tendon and patella [30]. These techniques employ local tissue to bridge the gap and include the Scuderi and Codivilla techniques. But it’s important to note that these techniques are dependent on tendon tissue that is frequently attenuated and of inferior quality, and the natural tendon is unavoidably weakened by the procedure. Therefore, we usually do not endorse the use of such techniques [31].

There are several graft options available for augmentation of the quadriceps tendon, such as autografts, allografts, or synthetic grafts. Autograft options may include the semitendinosus or gracilis tendon [32]. In the case of allografts, various options such as the Achilles, tibialis anterior, and semitendinosus tendons have been utilized previously with reports of bone-tendon allograft use, especially in cases of chronic ruptures or failed repairs [31]. While allograft tissues offer the benefit of avoiding donor-site morbidity and enabling implantation of a large graft [33], there is some risk of infectious disease transmission associated with their use. Additionally, there can be delayed biologic incorporation of allograft tissue, potentially due to an immune response to the foreign tissue [34].

Therefore the choice of surgical technique depends on the extent and quality of the tendon tissue, as well as the patient’s underlying medical conditions and physical abilities.

Rehabilitation after surgery is critical for a successful outcome, which typically involves a combination of physical therapy and progressive weight-bearing exercises. Published literature on rehabilitation protocols following surgical repair of ruptured tendons recommends a period of restricted weight bearing or flexion for at least 6 weeks [35, 36]. However, early initiation of passive motion has been found to facilitate the healing process and yield several benefits, including increased tensile strength, improved gliding function, and superior joint mechanics when compared to immobilized tendons [37]. More aggressive protocols have been described with full weight bearing allowed in a brace locked in extension for 6 weeks while allowing early active knee flexion up to 55° a few days postoperatively, and this protocol was associated with good outcomes [38]. A comparative study evaluating the effectiveness of an aggressive rehabilitation protocol, which allowed passive and active knee flexion in a controlled manner with a hinge knee brace as follows The brace was set to 30° for 2 weeks, then increased to 60° for weeks 2–4, and then to 90° for weeks 4–6 postoperatively, with immediate full weight bearing. The study demonstrated that such an approach is safe and does not result in inferior clinical outcomes or an increased incidence of complications, as compared to more restrictive protocols [39].

## Conclusion

In conclusion, bilateral spontaneous quadriceps tendon rupture is an uncommon condition and the likelihood of misdiagnosis during initial evaluation is high due to factors such as obesity, medical conditions, and insufficient examination. As a result, it is imperative to conduct a thorough medical evaluation that encompasses a detailed medical history, physical examination, and imaging to ensure a precise diagnosis and timely intervention as early surgical repair showed to be more favorable for better prognosis as compared to delayed repair [36, 40].
